# Chronic endometritis and recurrent implantation failure: a narrative review of clinical relevance and diagnostic challenges

**DOI:** 10.1530/RAF-25-0166

**Published:** 2026-06-08

**Authors:** Pavel Yakovlev, Darya Supranovich, Lyubov Yakovleva, Mariia Kornilova, Arina Kordys

**Affiliations:** ^1^ Centre for Reproductive Medicine, Next Generation Clinic, Moscow, Russia; ^2^ Centre for Reproductive Medicine, EVACLINIC IVF, Minsk, Belarus; ^3^ Chuvash State University named after I.N. Ulyanov, Cheboksary, Russia; ^4^ Mari State University, Yoshkar-Ola, Russia; ^5^ Wesleyan University, Middletown, Connecticut, USA

**Keywords:** chronic endometritis, recurrent implantation failure, CD138, plasma cells, endometrial biopsy, IVF

## Abstract

**Abstract:**

Chronic endometritis (CE) is a localized inflammatory disorder of the endometrium characterized by plasma cell infiltration, most often identified by CD138 immunostaining. Its diagnosis remains problematic due to the absence of standardized thresholds, variability in biopsy timing, and inconsistent histologic interpretation, which results in heterogeneous prevalence reports. CE has been proposed as a potential contributor to recurrent implantation failure (RIF); however, the available evidence remains limited. True RIF, defined as repeated implantation failure despite transfer of euploid embryos, affects only 2–5% of patients. Some recent comparative studies suggest that the prevalence of CE in women with RIF is not higher than in control populations. These findings indicate that the contribution of CE to implantation failure may be less prominent than previously assumed. Interpretation of existing data is further complicated by variability in diagnostic criteria, thresholds for plasma cell detection, biopsy timing, and study design. In this context of diagnostic uncertainty, management remains empirical, with antibiotics frequently prescribed without confirmed causative pathogens. This practice raises concern given the limited and inconsistent evidence of therapeutic benefit, the risk of overdiagnosis and overtreatment, and concerns regarding unnecessary antibiotic exposure and antimicrobial resistance. This narrative review synthesizes current evidence on the diagnosis, clinical relevance, and management of CE in the context of RIF and provides a clinical algorithm to guide selective screening and clinical decision-making. Overall, available data support a cautious, individualized, and selective approach. Further prospective studies are required to establish standardized diagnostic criteria and clarify whether treatment of CE improves reproductive outcomes.

**Lay summary:**

Chronic endometritis is a persistent inflammation of the inner lining of the uterus that has been suspected as a cause of repeated failure of embryo implantation during fertility treatment. However, diagnosis is challenging because there are no clear medical standards, and results vary between studies. Recent research shows that true repeated implantation failure affects only about 2–5% of patients, and chronic endometritis does not appear to be more common in these cases than in others. Despite this uncertainty, antibiotics are often prescribed without clear evidence of benefit. This may expose patients to unnecessary side effects and contributes to growing antibiotic resistance. This review summarizes current evidence and presents a clinical algorithm to guide careful and selective testing. A more cautious and individualized approach may help avoid unnecessary treatment and support safer, more effective fertility care.

## Introduction

In the past decade, there has been a steady increase in publications on the association of chronic endometritis (CE) with several gynecological conditions, including unexplained infertility, recurrent implantation failure (RIF), and recurrent pregnancy loss. This trend is accompanied by a rising frequency of CE diagnoses in clinical practice, followed by treatment, particularly in patients with infertility. Given the lack of universally accepted diagnostic criteria, there is a risk of misinterpreting research findings and overdiagnosing the condition. This can lead to the prescription of expensive and often unwarranted treatment regimens, iatrogenic complications, and loss of time, especially in the context of infertility therapy. This publication reviews current perspectives on the diagnosis and treatment of CE in patients with infertility, as well as the available evidence to date on the possible association of CE with RIF and the effectiveness of *in vitro* fertilization (IVF) protocols.

## Methods

The literature search was conducted using the following databases: MEDLINE, Google Scholar, Scopus, EMBASE, Global Health, and Web of Science. We searched these databases for studies published in English up to August 2025. The search employed combinations of the following Medical Subject Headings and relevant keywords in various orders: ‘endometritis’, ‘chronic’, ‘diagnosis’, ‘immunohistochemical’, ‘hysteroscopy’, ‘infertility’, ‘IVF’, ‘pathophysiology’, and ‘recurrent implantation failure’. The reference lists of included studies were also screened to identify additional studies not captured by the electronic search. Priority was given to original research and certain review articles published within the last five years. All articles were available in full-text format. The data were synthesized as a narrative literature review. In addition, a recently published meta-analysis relevant to the topic (Newnham *et al.* 2026) was included during the revision process to ensure up-to-date evidence.

## Definition

There is no universally accepted definition of this condition. Chronic endometritis is a persistent inflammatory condition of the endometrium, associated with an immune response to bacterial pathogens and histologically characterized by plasma cell infiltration of the endometrial stroma (Cicinelli *et al.* 2008, Kitaya & Yasuo 2011, Moreno *et al.* 2018, Liu *et al.* 2019).

## Etiology

The primary cause of CE is a microbial infection of the uterine cavity, which leads to a chronic inflammatory process in the endometrium (Cicinelli *et al.* 2008, 2021). In this condition, the number of lactobacilli in the uterine cavity may decrease along with the increasing prevalence of bacterial pathogens (Liu *et al.* 2019). Several studies have reported bacterial species frequently associated with CE, including *Streptococcus spp., Escherichia coli, Enterococcus faecalis, Staphylococcus *spp*., Mycoplasma hominis, Ureaplasma urealyticum, Klebsiella pneumoniae, *and* Gardnerella vaginalis* (Cicinelli *et al.* 2008, 2025, Moreno *et al.* 2018). While *Chlamydia trachomatis* and *Neisseria gonorrhoeae* are the main pathogens responsible for acute endometritis, they are rarely implicated in CE (Cicinelli *et al.* 2008, Moreno *et al.* 2018).

## Prevalence

The lack of universally accepted diagnostic criteria makes it difficult to determine the true prevalence of the disease. The prevalence of CE in the general population is unclear, whereas among women with infertility, it ranges from 2.8–13% (Kasius *et al.* 2011, Liu *et al.* 2018, Herlihy *et al.* 2022, HogenEsch *et al.* 2023, Yilmaz *et al.* 2025) up to 56.8% (Cicinelli *et al.* 2018). CE is identified in up to 29.67% of patients experiencing recurrent pregnancy loss (Pirtea *et al.* 2021*a*). Women at increased risk of CE development include those presenting with prolonged menstrual bleeding episodes, an abortion history, fallopian tube obstruction (Chen *et al.* 2016), a cesarean scar defect (Wei *et al.* 2022), and the presence of endometrial polyps and intrauterine adhesions (synechiae) within the uterine cavity (Kuroda *et al.* 2022). CE is more frequently observed in women with endometriosis (Kalaitzopoulos *et al.* 2025).

## Diagnosis

### Clinical manifestations

CE has no specific clinical features (Heatley 2004, Pitsos *et al.* 2009, Smith *et al.* 2010). Some researchers consider abnormal uterine bleeding, pelvic pain, and dyspareunia as possible signs of CE (Kitaya & Yasuo 2011). However, these symptoms are nonspecific (Pitsos *et al.* 2009, Smith *et al.* 2010), and their severity does not correlate with the number of endometrial plasma cells in diagnosed CE (Heatley 2004, Smith *et al.* 2010).

The diagnosis of CE has traditionally been made by identifying plasma cells within the endometrial stroma (Greenwood & Moran 1981, Margulies *et al.* 2021). Plasma cells can be detected by histological staining with hematoxylin and eosin (H&E) or by immunocytochemical methods targeting syndecan-1 (CD138), a surface proteoglycan specific to plasma cells (Inki 1997, Kitaya & Yasuo 2013, Margulies *et al.* 2021).

Immunohistochemical evaluation of CD138+ cells is of primary importance for the diagnosis of CE in clinical practice (Liu *et al.* 2018, Herlihy *et al.* 2022), as it provides the highest accuracy and minimal bias compared with histological assessment by microscopy (Kitaya & Yasuo 2013). However, the number of CD138+ cells required for the diagnosis of CE has yet to be established (Li *et al.* 2021, Herlihy *et al.* 2022, Vitagliano *et al.* 2022). To date, there are no universally accepted standards for the diagnosis of CE (Margulies *et al.* 2021, Herlihy *et al.* 2022); consequently, the diagnostic criteria based on CD138 expression vary widely across different studies (Cicinelli *et al.* 2018, Li *et al.* 2021, Margulies *et al.* 2021). Furthermore, there are several limitations of immunohistochemical analysis in the diagnosis of CE (Table 1):

- Endometrial glandular epithelial cells express CD138 on their basal surface during the proliferative phase of the menstrual cycle. Monoclonal antibodies directed at CD138 on plasma cells may cross-react with this antigen on endometrial epithelial cells, but the resulting immunostaining is usually less intense compared with plasma cells (Inki 1997).
- Standardized protocols and conditions for immunohistochemical evaluation of CD138 in human endometrium have not yet been established (Margulies *et al.* 2021). The diagnosis may be influenced by various laboratory factors, including antibody selection, dilution, detection systems, and analytical conditions (Torlakovic *et al.* 2015).
- The technique used to collect endometrial tissue, the equipment involved in biopsy, sample processing, and analysis, are additional crucial factors that may impact the accuracy of CE diagnosis. Plasma cells may be diffusely spread within the endometrial stroma or be concentrated in patches, and in some cases, these cells might be missed in small biopsy samples (Kitaya & Yasuo 2011). In addition, in certain cases of CE, plasma cells are only found in the basal layer of the endometrium (Kitaya & Yasuo 2011).
- The number of CD138+ plasma cells varies during the menstrual cycle (Song *et al.* 2018, Ryan *et al.* 2022). The result may vary significantly depending on the day of the menstrual cycle on which the endometrial biopsy is performed.

**Table 1:** Limitations of immunohistochemical analysis in diagnosing chronic endometritis.

Factor	Limitations
CD138 expression by endometrial epithelial cells (Inki 1997)	Monoclonal antibodies to CD138 cross-react with epithelial cells, causing less intense but potentially confounding staining
Lack of standardized immunohistochemistry protocols (Torlakovic *et al.* 2015)	Variability in antibody choice, dilution, incubation time, and detection systems affects diagnosis consistency
Tissue collection technique and biopsy equipment (Kitaya & Yasuo 2011)	Sampling error may either miss focal plasmacytosis or overrepresent it depending on the biopsy site, leading to overdiagnosis or, conversely, to a false-negative result
Menstrual cycle phase during biopsy (Song *et al.* 2018, Ryan *et al.* 2022)	CD138+ plasma cell counts fluctuate during the cycle, so biopsy timing significantly influences results

Accordingly, a cautious approach is recommended for both the use of this method and the interpretation of its results. The study by Miguel *et al.* demonstrated that CD138+ cells were absent in 25% of endometrial biopsies in which plasma cells were identified using histological staining (Miguel *et al.* 2011). Conversely, in 35% of samples marked as CD138+, no signs of CE were detected upon evaluation with conventional staining (Miguel *et al.* 2011). Therefore, in the search for reliable diagnostic methods for CE, some authors emphasize, alongside the detection of CD138+ cells, the importance of direct histological assessment of stromal changes in the endometrium, such as spindling of cells, edema, breakdown, pigment deposition, areas of hypercellularity, and the presence of inflammatory cells other than plasma cells (lymphocytes, eosinophils, neutrophils, and histiocytes) (McQueen *et al.* 2021). In terms of histologic criteria, Murdock’s Diagnosis of Endometrial Biopsies and Curettings provides detailed morphologic standards that aid in distinguishing true inflammation from nonspecific stromal changes and in highlighting potential diagnostic pitfalls (Murdock *et al.* 2019). The combination of histologic examination and immunohistochemistry may reduce false-positive diagnoses of CE (McQueen *et al.* 2021). Other researchers have adopted comprehensive diagnostic strategies that correlate CD138-positive cells with concomitant stromal features (HogenEsch *et al.* 2023).

### Timing of endometrial biopsy and its influence on CE diagnosis

There is no clearly established optimal timing within the menstrual cycle for performing endometrial biopsy for the diagnosis of CE. In some studies, endometrial biopsy was performed during the follicular phase (Cicinelli *et al.* 2008, 2021, Chen *et al.* 2016), in others, in the periovulatory period (Herlihy *et al.* 2022) or during the luteal phase (Liu *et al.* 2018, Li *et al.* 2021, Yilmaz *et al.* 2025) of the menstrual cycle. Plasma cells in women with CE are more commonly found in the early follicular phase (days 5–8) than in the late follicular (days 9–14) or luteal phases (Song *et al.* 2018, Ryan *et al.* 2022). In this context, Li *et al.* demonstrated that endometrial CD138+ cell levels were significantly higher in the proliferative phase than in the mid-luteal phase in the same patients, further underscoring the impact of biopsy timing on CE detection (Li *et al.* 2025). Therefore, sampling during the luteal phase may help reduce the risk of CE overdiagnosis. For example, Liu *et al.* identified signs of CE in only 10.4% of infertile women and in 5% of fertile women when detecting CD138+ cells in endometrial tissue seven days after the luteinizing hormone surge in the blood (Liu *et al.* 2018). Similarly, in the study by Yilmaz *et al.*, all samples were obtained during the mid-luteal phase, which may have contributed to a lower detection rate compared with studies using proliferative-phase samples (Yilmaz *et al.* 2025). A lower prevalence of CE in the presented study, compared with the literature data, may be related to the overdiagnosis of CE when endometrial tissue is sampled during the follicular phase of the cycle. Further studies are needed to investigate the optimal timing for performing endometrial biopsy to exclude CE.

### Criteria for diagnosing CE by immunohistochemistry

As noted above, no consensus exists on the plasma cell density threshold for diagnosing CE (Li *et al.* 2021, Herlihy *et al.* 2022, Vitagliano *et al.* 2022). It is known that a few CD138+ plasma cells can be found in endometrial samples from 30% of fertile women (McQueen *et al*. 2021). According to a survey of members of international pathology societies, 28.5% of pathologists diagnose CE based on the presence of a single plasma cell, 35% on 2–5 cells, 18% on more than 5 cells, and 16% on the detection of a cell cluster (starting from three plasma cells) in the evaluated specimen (Margulies *et al.* 2021).

Establishing a CD138+ threshold for initiating treatment of CE remains challenging; equally important is determining whether such thresholds exert a clinically meaningful effect on reproductive outcomes.

Herlihy *et al.* examined endometrial biopsies from 80 IVF patients undergoing single, euploid blastocyst transfer and found plasma cell infiltration in almost half of the samples: 49% contained at least one plasma cell, whereas only 11% showed five plasma cells per 10 high-power fields (HPFs), and just 4% displayed one plasma cell in every HPF. When comparing clinical pregnancy rate (CPR) and live birth rate (LBR) after euploid embryo transfer, no statistically significant differences were found between patients with one plasma cell per 10 HPFs and those with none, suggesting that this threshold may lead to overdiagnosis of CE. Although women with ≥5 plasma cells per 10 HPFs showed lower LBR, the difference was not statistically significant (Herlihy *et al.* 2022).

A 2023 meta-analysis by Santoro *et al.* of nine observational studies found no significant association between plasma cell cut-offs and pregnancy or live birth rates. A significant association with miscarriage emerged only at higher plasma cell burdens: the risk increased when counts reached ≥5 cells per 10 HPFs (RR: 2.4; *P* = 0.007). In individual studies excluded from quantitative pooling, an adverse impact on pregnancy appeared solely when even stricter thresholds – 10 or 50 cells per 10 HPFs – were applied (Santoro *et al.* 2023). The authors emphasized the heterogeneity of included studies and the limited evidence for establishing a definitive plasma cell threshold (Santoro *et al.* 2023).

The 2022 meta-analysis by Vitagliano *et al.* indicated that CE adversely affects assisted reproductive technology (ART) outcomes only in its severe form – characterized by at least five plasma cells per HPF – whereas mild disease (one to four plasma cells per HPF) does not correlate with reduced embryo implantation (Vitagliano *et al.* 2022).

In a propensity score-matched cohort study by Xu *et al.*, patients with mild CE (1–4 CD138+ plasma cells/HPF) undergoing frozen-thawed embryo transfer showed no significant differences in LBR, CPR, miscarriage rates, or perinatal outcomes between antibiotic-treated and untreated groups, and higher CD138 counts had no prognostic impact (Xu *et al.* 2025).

### Hysteroscopy

Possible hysteroscopic markers of CE include endometrial hyperemia, the presence of micropolyps, and endometrial interstitial edema (Song *et al.* 2019, Liu *et al.* 2020). The accuracy of hysteroscopy in diagnosing CE is only 67%, so this method is not recommended as a substitute for histologic examination (Song *et al.* 2019). According to Liu *et al.*, the sensitivity and specificity of diagnosing CE by hysteroscopy are 62.8 and 91.7%, respectively (Liu *et al.* 2020). It should also be noted that the diagnostic value of hysteroscopy depends directly on the expertise and experience of the physician performing the procedure.

The International Working Group for the Standardization of CE Diagnosis proposed the following hysteroscopic diagnostic criteria for CE (Cicinelli *et al.* 2019):

- Strawberry aspect – broad, intensely hyperemic areas dotted with white central points.
- Focal hyperemia – small, sharply demarcated red patches.
- Hemorrhagic spots – pinpoint or irregular red areas, sometimes contiguous with superficial capillaries.
- Micropolyps – ≤1 mm papillary projections with a central vascular core scattered singly or diffusely.
- Stromal edema giving the endometrium a thick, pale appearance when observed in the follicular phase (a normal finding during the secretory phase) (Cicinelli *et al.* 2019).

## Treatment

Given that the etiology of CE is linked to bacterial infection (Liu *et al.* 2019, Cicinelli *et al.* 2021), the proposed treatment should include only the administration of antibacterial agents. The use of alternative methods lacks evidence of efficacy.

Endometrial sampling can identify pathogens responsible for CE, thereby facilitating precisely targeted antibiotic therapy (Cicinelli *et al.* 2018). However, the causative agents of CE cannot always be identified (Moreno *et al.* 2018). The uterine cavity is not sterile, and the presence of microorganisms alone does not necessarily indicate inflammation (Cicinelli *et al.* 2009, Mitchell *et al.* 2015). Moreover, vaginal and intrauterine microbiota do not coincide in most cases (Cicinelli *et al.* 2008, 2009, Mitchell *et al.* 2015), and microbial studies using samples from the lower genital tract cannot predict the pathogens responsible for CE (Cicinelli *et al.* 2009, Mitchell *et al.* 2015). Therefore, in most cases, the treatment of CE involves empirically chosen antibiotics, despite the fact that inappropriate treatment may lead to recurrence of CE or drug resistance. First-line therapy typically consists of doxycycline (100 mg twice daily for 10–14 days), which remains the most commonly recommended initial regimen. If CE persists on follow-up biopsy, second-line treatment may include combination antibiotic therapy, most commonly ciprofloxacin with metronidazole, which has demonstrated effectiveness in achieving histologic resolution in a substantial proportion of persistent cases. There is limited evidence regarding third-line treatment for CE; however, amoxicillin–clavulanate may be considered given its broad antimicrobial spectrum and reported efficacy (Strug *et al.* 2024).

### Antimicrobial therapy monitoring

The utility of performing a confirmatory biopsy following therapy is under discussion. The 2022 study by Liu *et al.*, which analyzed ART outcomes in 1,261 patients diagnosed with CE, found no impact of endometrial re-examination on clinical ART outcomes in women with CE treated first-line with doxycycline (Liu *et al.* 2022). A systematic review and meta-analysis by Vitagliano *et al.* showed that antibiotic therapy without confirmation of cure does not improve ongoing pregnancy rate (OPR)/LBR or CPR; benefits were observed only in patients with documented eradication of CE on follow-up biopsy, with outcomes comparable to those without CE (Vitagliano *et al.* 2018). Similarly, a 2022 systematic review and meta-analysis by Cheng *et al.* concluded that antibiotic treatment improves pregnancy outcomes in RIF only when CE cure is confirmed by a control biopsy (Cheng *et al.* 2022).

## Antibiotic resistance

Kitaya *et al.* conducted an ambispective cohort study to determine the prevalence of antibiotic resistance in CE among women with RIF, defined as having at least three failed embryo transfers in their medical history. Resistance to first-line treatment for CE (i.e. oral doxycycline 200 mg/day for 14 days) was detected in 21.2% of cases. Multiple drug resistance (MDR) was defined as resistance to both first- and second-line treatments, the latter being defined as a combination of oral metronidazole (500 mg/day) and ciprofloxacin (400 mg/day) for 14 days. The prevalence of MDR in the treatment of CE increased 8.27-fold, from 1.3% (during 2010–2015) to 9.6% (during 2015–2020) (Kitaya *et al.* 2022).

In the same study, the efficacy of oral moxifloxacin (400 mg/day for 10 days) and oral azithromycin (500 mg/day for 3 days) was compared as empirical third-line antibiotic therapy for MDR CE in women with RIF, with cure rates of 79.2 and 75%, respectively (Kitaya *et al.* 2022). Moreover, resolution of persistent MDR CE was achieved in some women with RIF following a 14-day oral course of lincomycin hydrochloride hydrate at a dose of 1,500 mg/day (Kitaya & Ishikawa 2022).

The overall efficacy of standard antibiotic regimens remains relatively low: persistent CE was observed in 31.5% of treated patients (HogenEsch *et al.* 2023). This low cure rate suggests high levels of antibiotic resistance in CE cases when treated with first-line medications (HogenEsch *et al.* 2023).

A recent study by Cicinelli *et al.* revealed increased resistance to first-line empirical CE therapies: tetracyclines (75.8%), quinolones (68.4%), and nitroimidazoles (39.3%). Macrolides remain the only class with preserved susceptibility (resistance 2.9%); however, their narrow spectrum of action limits clinical application. A significant correlation was also established between patient age and the frequency of extended-spectrum beta-lactamase producers and penicillin resistance, which may reflect the cumulative impact of previous antibiotic therapy and age-related changes in immune status (Cicinelli *et al.* 2025).

These findings highlight the common occurrence of antibiotic resistance in CE treatment. Given that CE is not a life-threatening condition and its precise role in infertility pathophysiology requires further clarification, clinicians should adopt a judicious approach to antibiotic prescription in these cases.

## Recurrent implantation failure and chronic endometritis in ART

There is currently no universally accepted definition of RIF. It is generally defined as the failure to achieve a clinical pregnancy after two to three transfers of morphologically high-grade, euploid embryos. The European Society of Human Reproduction and Embryology (ESHRE) recommends a cumulative predicted implantation probability of 60% as the threshold for defining RIF and initiating further diagnostic workup (ESHRE Working Group on Recurrent Implantation Failure *et al.* 2023). The woman’s age at the time of oocyte retrieval and the embryo’s chromosomal status following preimplantation genetic testing for aneuploidy (PGT-A) must be taken into account (ESHRE Working Group on Recurrent Implantation Failure *et al.* 2023).

Recent evidence suggests that the actual incidence of RIF ranges between 2 and 5%. These findings are supported by studies demonstrating high cumulative success rates following transfers of euploid embryos selected through PGT-A. Pirtea *et al.* demonstrated a 95% cumulative implantation rate following three subsequent euploid blastocyst transfers (Pirtea *et al.* 2021*b*). These findings are consistent with the report by Ata *et al.*, who demonstrated that, assuming an implantation rate of 55% per euploid blastocyst, transfer of three and four blastocysts is sufficient to achieve cumulative implantation probabilities exceeding 90 and 95%, respectively (Ata *et al.* 2021). In turn, R. B. Gill *et al.* demonstrated that the cumulative live birth rate (CLBR) in women with a history of RIF reaches 98.1% (95% CI: 96.5–99.6%) after five transfers of euploid embryos (Gill *et al.* 2024). The authors did not find a statistically significant decrease in the LBR with subsequent transfers. For patients with three prior failed transfers of euploid embryos, the LBR after the fourth and fifth consecutive transfers was 40 and 53.3%, respectively (Gill *et al.* 2024). The central role of embryonic factors is further confirmed by a 2026 meta-analysis demonstrating that PGT-A significantly increases both the LBR per transfer (OR 2.79 (95% CI: 1.90, 4.10)) and CLBR per cycle (OR 4.23 (95% CI: 2.14, 8.38)) in women with RIF (Newnham *et al.* 2026). A large retrospective cohort study by Dhaenens *et al.* demonstrated that CLBR continued to rise with each additional blastocyst transfer, reaching 78% after the tenth attempt, without evidence of a plateau even in the absence of PGT-A. Importantly, the cohort was not preselected to exclude women with uterine pathologies, implying that even in the presence of possible endometrial abnormalities, including CE, live birth remained achievable (Dhaenens *et al.* 2025). These data suggest that embryonic factors likely represent the predominant contributor in most cases of implantation failure, whereas the isolated contribution of endometrial pathology to implantation failures appears to be less pronounced.

The prevalence of CE among patients with RIF ranges from 6.35–7.7% (Liu *et al.* 2018, Ticconi *et al.* 2024, Yilmaz *et al.* 2025) to 12.4% (Wang *et al.* 2025). A 2024 systematic review and meta-analysis by Ticconi *et al.* examined potential links between CE, infertility, and RIF and reported that CE is significantly more common in infertile women compared with fertile women (19.46 vs 7.7%, *P* = 0.001). However, there was no difference in CE prevalence between patients with RIF and fertile controls (6.35 vs 5.8%, *P* = 0.9), suggesting no association between CE and RIF (Ticconi *et al.* 2024). In the study of Yilmaz *et al.*, the prevalence of CE was similar in infertile patients with and without prior implantation failure, at 7.9 and 6.3%, respectively; among patients with a history of three or more failed implantations, the prevalence of CE was only 2%, suggesting that CE is unlikely to be a major contributor to RIF (Yilmaz *et al.* 2025).

Evidence on CE and ART outcomes depends on population and methods. In ART cohorts not restricted to RIF, a 2022 meta-analysis reported no improvement in implantation, CPR, or LBR with oral antibiotics for CE (Kato *et al.* 2022). In RIF-focused syntheses, both Vitagliano *et al.* (2018) and Cheng *et al.* (2022) found that improved outcomes are observed only when CE cure is documented on a follow-up biopsy, whereas empiric antibiotic therapy without confirmation of eradication does not confer benefit; moreover, post-cure outcomes approximate those of CE-negative patients in pooled analyses (Vitagliano *et al.* 2018, Cheng *et al.* 2022). Across reviews, nonrandomized designs, heterogeneous CE diagnostic thresholds and CD138 practices, variable RIF definitions, and rebiopsy-related selection and verification biases limit causal inference and generalizability.

Based on the available evidence, the ESHRE guidelines for managing patients with RIF note that firm recommendations regarding the diagnostic and therapeutic value of CE cannot yet be established. Nevertheless, in patients with RIF, screening for CE and, if the diagnosis is confirmed, subsequent treatment are considered acceptable (ESHRE Working Group on Recurrent Implantation Failure *et al.* 2023). The 2020 guideline of the Canadian Fertility and Andrology Society does not recommend routine testing for CE in women with RIF due to the presence of small, low-quality, heterogeneous observational studies and the lack of consensus diagnostic criteria for CE (Shaulov *et al.* 2020).

There are currently no unequivocal recommendations to test for CE in patients with RIF. If CE represents a major independent contributor to implantation failure, one would expect its prevalence to be higher in patients with RIF than in control groups. However, this hypothesis was not supported by the 2024 systematic review and meta-analysis by Ticconi *et al.*, which demonstrated no significant difference in CE prevalence between women with RIF and fertile controls (6.35 vs 5.8%, *P* = 0.9) (Ticconi *et al.* 2024). These findings are consistent with the 2025 study by Yilmaz *et al.*, conducted in an infertility cohort, which reported comparable CE rates in infertile patients without prior implantation failure and those with a history of failed embryo transfer (6.3 vs 7.9%). Stratification by the number of previous failed transfers showed CE in 12.2% of cases after one failure, 7.4% after two failures, and 2.0% among women with three or more failed transfers. No statistically significant differences in CE prevalence were observed according to implantation history or number of prior failures (Yilmaz *et al.* 2025). Collectively, these data suggest that CE is unlikely to represent a major cause of implantation failure. In light of this evidence, the uncertain association between CE and RIF, and the substantial heterogeneity of available studies – including variability in diagnostic criteria, CD138 cutoff thresholds, and biopsy timing – it is reasonable to minimize routine diagnostic testing and empirical antibiotic treatment, restricting their use primarily to the framework of scientific research until robust evidence of clinical benefit becomes available. Against this background, the widespread use of empirical antibiotics warrants particular caution, given the limited and inconsistent evidence of therapeutic benefit, the risk of overtreatment, and growing concerns regarding antimicrobial resistance, underscoring the critical need for strict antimicrobial stewardship in reproductive medicine.

## Proposed clinical management strategy

CE has been postulated as a potential contributor to implantation failure. However, the lack of consensus on its diagnostic criteria makes it difficult to study and understand when a CE diagnosis has true clinical significance. Given the low prevalence of true RIF (2–5%) and the available evidence indicating that CE is not more common in well-defined RIF populations than in appropriate control groups, routine CE evaluation after a failed embryo transfer appears unlikely to provide substantial clinical benefit. In the context of diagnostic uncertainty and heterogeneous study designs, indiscriminate testing may increase the risk of overdiagnosis and unnecessary antibiotic exposure without clear improvement in reproductive outcomes. This uncertainty is further compounded by significant variability in diagnostic approaches and interpretation of histopathological findings, which continues to challenge the clinical applicability of CE testing. This lack of uniformity is primarily driven by three key methodological and biological challenges:

- There is no consensus on the diagnostic criteria for CE. Diagnosis may rely on H&E staining, CD138 immunohistochemistry, or hysteroscopic findings, yet even experienced pathologists may have difficulty distinguishing plasma cells from other stromal cells, and no universally accepted CD138-positive cell threshold has been established.
- Plasma cell density varies across the menstrual cycle, and biopsy timing may substantially influence detection rates, potentially contributing to inconsistent prevalence estimates across studies.
- The clinical relevance of low-level plasma cell infiltration remains uncertain. Some studies report no association between low CD138 thresholds and impaired implantation, whereas others suggest that only higher plasma cell burdens may be clinically meaningful, and proposed treatment cutoffs differ considerably.

Therefore, a more structured and clinically reasoned approach to CE assessment is warranted. Based on the available evidence and the absence of clear consensus, we propose the following clinical considerations for the evaluation of CE in patients with RIF:

- Diagnostic and therapeutic decisions regarding CE should be made cautiously, taking into account the limited quality and reproducibility of the available evidence.
- Routine CE testing may not be justified in all patients with infertility or prior to IVF, particularly when performed solely to increase implantation likelihood, as current data do not demonstrate consistent clinical benefit and may expose patients to unnecessary interventions.
- In patients with RIF, CE testing should not be performed indiscriminately and is best reserved for selected cases, given the uncertain association with implantation failure and the risk of overdiagnosis.
- When CE testing is undertaken, clinicians may consider prioritizing CD138 (syndecan-1) immunohistochemistry, alone or in combination with conventional histologic evaluation, as it may improve diagnostic consistency and reduce the risk of misinterpretation compared with H&E staining alone.
- Endometrial biopsy, if performed, may be considered in the mid-luteal phase, as cycle-dependent variation in plasma cell density may otherwise increase the likelihood of overdiagnosis.
- Low-level plasma cell findings should be interpreted with caution, as they may be observed in fertile women and do not necessarily indicate clinically meaningful inflammation; antibiotic treatment should therefore not be routinely initiated in such cases.

**Figure 1:**
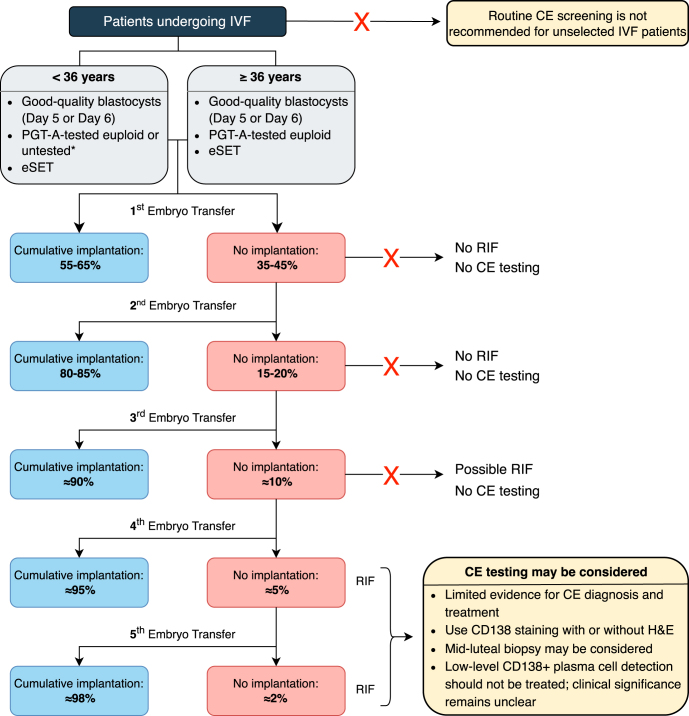
Proposed management algorithm for recurrent implantation failure based on sequential embryo transfers and selective chronic endometritis screening. Projected cumulative implantation rates following sequential PGT-A-tested euploid embryo transfers are derived from published studies (Ata *et al.* 2021, Gill *et al.* 2024). *In women <36 years, routine PGT-A is not recommended. Due to the high prevalence of euploidy in this population, morphologically good-quality untested blastocysts possess high reproductive potential, supporting a similar sequential transfer approach. eSET, elective single embryo transfer; PGT-A, preimplantation genetic testing for aneuploidy; RIF, recurrent implantation failure; CE, chronic endometritis; H&E, hematoxylin and eosin; CD138, immunohistochemical staining for syndecan-1.

Figure 1 presents a proposed diagnostic algorithm for the evaluation of CE in patients with RIF, designed to minimize embryonic confounding and to allow focused assessment of endometrial factors. Accurate diagnosis of RIF requires the exclusion of structural uterine pathology via ultrasound and should be based on outcomes following the transfer of embryos with high developmental competence. To reduce embryonic confounding, blastocyst-stage transfers are preferred when evaluating implantation potential, as cleavage-stage embryos have significantly lower implantation rates. Because aneuploid embryos are strongly associated with implantation failure, the proposed algorithm is primarily intended for sequential transfers of euploid blastocysts following PGT-A. Recent evidence suggests that the use of PGT-A in patients with unexplained RIF is associated with a statistically significant increase in LBR, supporting the hypothesis that RIF may represent an embryological phenomenon (Newnham *et al.* 2026). However, since PGT-A has not consistently been shown to improve clinical outcomes in the general IVF population, while demonstrating benefit primarily in women of advanced reproductive age (Munné *et al.* 2019, Simopoulou *et al.* 2021, Yan *et al.* 2021, Mejia *et al.* 2022), patients were stratified by age (<36 and ≥36 years). Nevertheless, the application of PGT-A in the advanced reproductive age group should be individualized and may not be appropriate or practical in all cases, particularly in those with diminished ovarian reserve. Although the cumulative implantation probabilities presented in the figure are derived from transfers of euploid embryos, the transfer of morphologically good-quality blastocysts without PGT-A was considered acceptable in women <36 years of age for the purpose of estimating implantation probability and defining RIF. This approach reflects the high prevalence of euploid embryos and comparable reproductive outcomes observed in younger women, even in the absence of routine PGT-A. Importantly, blastocyst morphology and developmental timing remain independent predictors of implantation, as higher inner cell mass and trophectoderm quality and earlier blastocyst formation (day 5–6 vs day 7) are associated with better outcomes, even among euploid embryos (Hernandez-Nieto *et al.* 2019, Reshef *et al.* 2022). Based on cumulative outcome data, true RIF may be more confidently suspected after multiple consecutive failed transfers of good-quality euploid blastocysts, particularly when the expected cumulative implantation probability exceeds approximately 90–95%, although consensus definitions vary and no universally accepted threshold has been established. This approach may reduce premature investigation and unnecessary interventions after fewer failed transfers, where continued success remains likely. Under these conditions, approximately 2–5% of patients would be expected to meet criteria consistent with true implantation failure. In this highly selected subgroup, CE evaluation, if performed, should follow a cautious and selective approach to minimize overdiagnosis and unnecessary treatment.

## Conclusion and future perspectives

Chronic endometritis remains a poorly defined inflammatory condition with uncertain clinical relevance in implantation failure. Current evidence suggests that embryonic factors likely play a predominant role in most cases of recurrent implantation failure, whereas the contribution of endometrial pathology appears limited. Given the lack of standardized diagnostic criteria and consistent evidence of benefit, routine screening and empirical antibiotic treatment for CE cannot be recommended. A cautious, selective, and individualized approach is warranted until high-quality prospective data clarify its clinical significance and therapeutic impact.

## Declaration of interest

The authors declare that there is no conflict of interest that could be perceived as prejudicing the impartiality of the work reported.

## Funding

This research did not receive any specific grant from any funding agency in the public, commercial, or not-for-profit sector.

## Author contribution statement

PY contributed to the concept and design of the investigation and editing; DS contributed to the concept and design of the investigation, material collection and processing, and writing the text; LY contributed to editing; MK contributed to writing the text and language and style editing; and AK contributed to material collection and processing and writing the text.
